# Recent advances in recombinase polymerase amplification: Principle, advantages, disadvantages and applications

**DOI:** 10.3389/fcimb.2022.1019071

**Published:** 2022-11-28

**Authors:** Meiying Tan, Chuan Liao, Lina Liang, Xueli Yi, Zihan Zhou, Guijiang Wei

**Affiliations:** Center for Clinical Laboratory Diagnosis and Research, The Affiliated Hospital of Youjiang Medical University for Nationalities, Baise, China

**Keywords:** isothermal amplification, recombinase polymerase amplification, pathogenic microorganism, genetically modified food, SARS-CoV-2

## Abstract

After the outbreak of SARS-CoV-2, nucleic acid testing quickly entered people’s lives. In addition to the polymerase chain reaction (PCR) which was commonly used in nucleic acid testing, isothermal amplification methods were also important nucleic acid testing methods. Among several common isothermal amplification methods like displaced amplification, rolling circle amplification, and so on, recombinase polymerase amplification (RPA) was recently paid more attention to. It had the advantages like a simple operation, fast amplification speed, and reaction at 37-42°C, et al. So it was very suitable for field detection. However, there were still some disadvantages to RPA. Herein, our review mainly summarized the principle, advantages, and disadvantages of RPA. The specific applications of RPA in bacterial detection, fungi detection, virus detection, parasite detection, drug resistance gene detection, genetically modified food detection, and SARS-CoV-2 detection were also described. It was hoped that the latest research progress on RPA could be better delivered to the readers who were interested in RPA.

## Introduction

Among the nucleic acid-based molecular diagnostic methods, the polymerase chain reaction (PCR) invented in the 1980s (Mullis et al., 1986) was the most widely used. However, due to the excessive dependence of PCR on temperature control equipment, time-consuming, and professional operation, it was difficult to apply PCR in on-site detection (Yan et al., 2014). In 2006, Piepenburg et al. developed a isothermal amplification method named by recombinase polymerase amplification (RPA) (Piepenburg et al., 2006). Due to its advantages like a simple operation, fast amplification speed, reaction at 37-42°C and, so on (Li et al., 2018), it was expected to replace PCR. Since RPA was established over ten years, it had been widely used in various fields like the detection of bacteria, fungi, parasites, viruses, drug resistance genes, and so on. Nowadays, through the continuous improvement of the sample treatment process, amplification system, and result detection system, RPA seemed to be more and more popular in molecular diagnosis. In particular, the outbreak of SARS-CoV-2 in 2019 further promoted the application of RPA in nucleic acid detection (Bai et al., 2022). Our review summarized the latest RPA research in the past five years that mainly cover several fields, including the detection of bacteria, fungi, parasites, viruses, drug resistance genes, genetically modified food, and SARS-CoV-2. It was hoped to provide a reference for further study of RPA.

## The principle of RPA

### The principle of basic RPA

**Figure 1:**
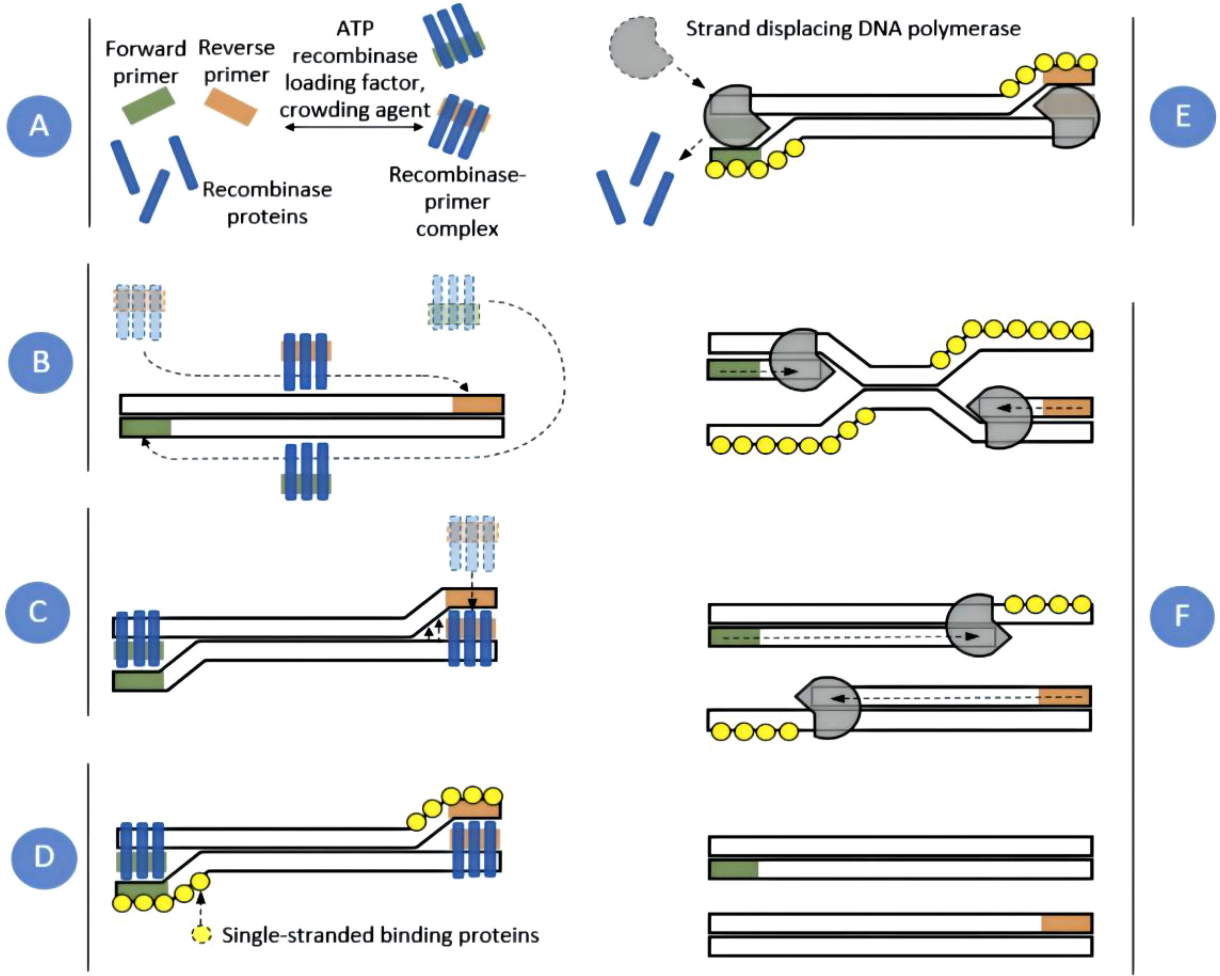
RPA amplification scheme. Recombinase proteins formed complexes with each primer **(A)**, which scanned DNA for homologous sequences **(B)**. The primers were then inserted at the cognate site by the strand-displacement activity of the recombinase **(C)** and single-stranded binding proteins stabilized the displaced DNA chain **(D)**. The recombinase then disassembled leaving the 3` end of the primers accessible to a strand displacing DNA polymerase **(E)**, which elongated the primer **(F)**. Exponential amplification was achieved by cyclic repetition of this process (Lobato and O’Sullivan, 2018).

RPA technology mainly includes two enzymes: recombinant enzyme T4 UvsX and bacillus subtilis Pol I (Piepenburg et al., 2006). Moreover, the RPA reaction system also needed amplification templates, primers, and various raw materials. The basic reaction process of RPA was as follows: First, in the presence of ATP and polyethylene glycol, the recombinase protein UvsX combined with RPA primers to form a recombinase-primer complex. Then, the complex could find the homologous sequence in the double-strained DNA template. Once the homologous sequence was found, it would insert into the template chain to form a D-ring structure and start the chain replacement reaction. To prevent the inserted primer from being expelled through branch migration, the replaced template chain was bound to the Single-stranded binding protein to maintain the stability of the single chain. Finally, the recombinase was isolated from the complex. In the presence of dNTPs, DNA polymerase was bound to the 3`-OH end of the primer for chain elongation to form a new complementary chain. Repeat the above steps to achieve exponential amplification of the target region on the template. The whole RPA process was very fast. Generally, detectable amplification products could be obtained in about 20 min. The basic principle of RPA was shown in **Figure 1** (Lobato and O’Sullivan, 2018). The RPA amplification products could be displayed by conventional agarose gel electrophoresis. In addition, the commonly used RPA methods were mainly exo-RPA and LFS-RPA.

### The principle of exo-RPA

**Figure 2:**
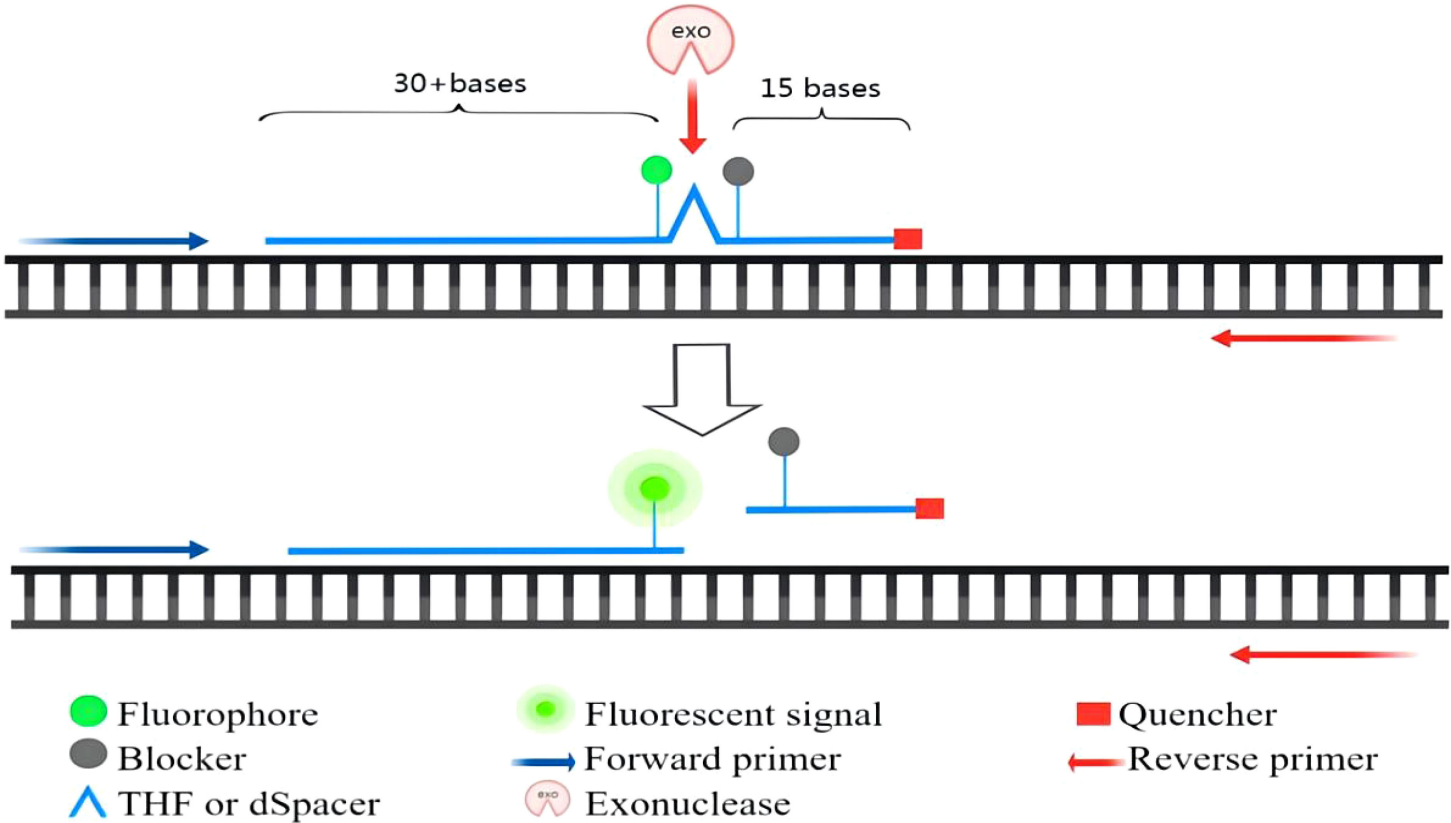
The principle of exo probe detection.

Real-time RPA was an assay that combines RPA with a fluorescent probe. This method allowed for the rapid detection of target genes while allowing real-time monitoring of the amplification process. According to the probe design requirements of the TwistAmp™Exo kit, a segment of sequence with a length of about 46-52 bases was first selected, and then a probe was synthesized according to the principle of complementary pairing. The probe was labeled by a fluorophore and had a quencher near the fluorophore to temporarily block the fluorescent signal. There was a blocker at the 3′ end that was used to prevent the polymerase from extending from the 3′ end. Real-time detection was based on the cleavage of the fluorescent probe at the abasic site between the fluorophore and the quencher. Abasic site could be tetrahydrofuran (THF) or a dSpacer (a derivative of the THF). The E. coli exonuclease III cleaved the probe at THF or dSpacer site, separating the fluorophore and quencher, thus releasing the fluorescent signal (Li et al., 2018). The fluorescence amplification curve could be obtained by continuously collecting fluorescence. The principle of exo probe detection was shown in **Figure 2**.

### The principle of LFS-RPA

**Figure 3:**
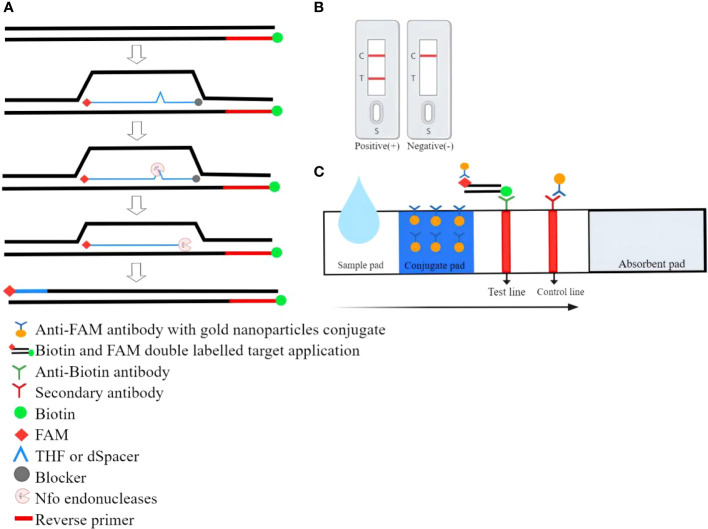
The working principle of RPA combined with lateral flow strip (LFS) assay. **(A)** During LFS-RPA, the reverse primer carries the biotin at the 5’ end and a modified probe was added to the reaction. Both ends of the probe were labeled with FAM and blocker respectively. Only when the probe was fully bound to the homologous sequence the endonuclease was able to cut the DNA double-strand and release the blocker. Thereby polymerase extended the substrate and chain synthesis continues leading to double-labeled amplicons. **(B)**Positive/negative results. **(C)**Schematic diagram of the RPA combination with lateral flow strip.

The RPA combined with lateral flow strip assay (LFS-RPA) was a method that combined basic RPA amplification technology, test strips, and immunoassay technology to enable visual detection. The principle of LFS-RPA detection was as follows: A sequence of about 46-52 bases in length was selected and a corresponding sequence was synthesized according to the principle of complementary pairing. The THF(or dSpacer) site was labeled in the middle of the sequence, carboxyfluorescein(FAM) and blocker were labeled at both ends, and biotin was labeled at the 5 ‘end of the reverse primer. During amplification, the Nfo endonucleases recognized the THF(or dSpacer) site and cleaved it, a double-stranded DNA labeled with FAM on one end and biotin on the other end was obtained. During LFS chromatography, RPA amplification products with colloidal gold nanoparticles(AuNPs) were first produced when the sample flows through the conjugate pad because the FAM-RPA amplification products could bind to anti-FAM antibodies with AuNPs. As the RPA amplified fragments moved forward with the AuNPs, their biotin group could bind to the anti-biotin antibody on the test line. The test line turned red due to the accumulation of AuNPs (Wang B et al., 2021). Colloidal gold complexes not captured by the anti-biotin antibody were captured by the secondary antibody on the control line, showing a red line indicating the validity of the LFS (Wang F et al., 2021a). The working principle of RPA combined with lateral flow strip (LFS) assay was shown in **Figure 3**.

## Comparison of RPA and PCR, other isothermal amplification methods

### Comparison of RPA and PCR

RPA, which emerged in 2006, was a new nucleic acid amplification technology that claims to replace PCR. Compared with PCR, its biggest advantage was that it could perform isothermal amplification at 37-42°C. **Table 1** compared RPA and PCR.

**Table 1:** Comparison of RPA and PCR.

Method	RPA	PCR
Origin	2006	1980s
Enzyme	Recombinase; DNA polymerase	Taq enzyme
Temp.	37-42°C	95°CDenaturation-55°Cannealing-72°Cextension
Time	5-20 min	1.5-2h
Number of primers	2	2
Reagent	Lyophilized or liquid	Liquid
Product detecting methods	Gel electrophoresis, real-time fluorescence, colloidal gold	Gel electrophoresis, real-time fluorescence, colloidal gold
Performance	Considerable sensitivity and specificity	High sensitivity and specificity
Advantage	Isothermal amplification; Short time; Low operation and instrument requirements Resistance to inhibitors; Tolerate more mismatches	Many conventional laboratories; Many commercial kits and wide applications; Mature technology; Reagents are relatively cheap; Ideal for quantification purposes
Disadvantage	High reagent price; There are few commercial kits, which is not conducive to large-scale detection; No special primer design software; Not used in clinical, only for scientific research; Prone to non-specific amplification; Poor quantitative separation rate	Need expensive instruments; The operation is highly specialized and requires special operation training; Intolerance to some substances
References	(Rohrman and Richards-Kortum, 2015; Oliveira et al., 2021; Xu et al., 2021; Munawar, 2022)	(Schrader et al., 2012; Oliveira et al., 2021; Munawar, 2022)

### Comparison of RPA and other isothermal amplification methods

In recent years, with the continuous development of nucleic acid-based amplification technology, the scientific community had been trying to find an isothermal amplification method that did not need PCR instruments. At present, the isothermal amplification technology that had been developed includes RPA, strand displaced amplification(SDA), rolling circle amplification(RCA), helicase-dependent isothermal DNA amplification(HDA), nucleic acid sequence-based amplification(NASBA), and loop-mediated isothermal amplification(LAMP), et al. Among these isothermal amplification methods, RPA was a relatively simple method.

The comparison between RPA and other isothermal amplification techniques was shown in **Table 2**.

**Table 2:** Comparison of RPA and other common isothermal amplification methods.

Method	RPA	SDA	RCA	HDA	NASBA	LAMP
Template	DNA/RNA	DNA	DNA/RNA	DNA/RNA	RNA	DNA/RNA
Time	5-20min	1-2h	1h	0.5-2h	1.5-2h	1h
Temp.	37-42°C	37-60°C	37-65°C	60-65°C	41°C	60-65°C
Number of primers	2	4	2	2	2	4-6
Number of enzymes	2	2	2	2	3	1
Advantage	Short reaction time; Tolerance of certain mismatches; Simple primer design and support for multiplex amplification reactions	Mild reaction conditions; Rapid amplification	Easy exponential amplification; Locked probe can make it have high specificity	Constant temperature reaction; Simple reaction structure	High selectivity to RNA molecules, free from background DNA interference; No additional cDNA processing required	High specificity; Simple colorimetric detection; Resistance to inhibitors
Disadvantage	No special primer design software; High reagent prices; Fewer reagent kits;	Low amplification efficiency for long targets; Strong non-specific background reaction; Thermal denaturation is required at first	Low purity annular template makes it difficult to control connection efficiency; The template needs to be a single chain ring structure;	Complicated buffer optimization	The reaction components are complex and require many enzymes; Not suitable for DNA virus detection	The primer design is complex; Easy to produce nonspecific amplification
References	(Xu et al., 2021; Munawar, 2022)	(Walker et al., 1992; Walker, 1993)	(Xu et al., 2021; Gao et al., 2022)	(Yan et al., 2014; Barreda-Garcia et al., 2018)	(Simpkins et al., 2000; Honsvall and Robertson, 2017; Gao et al., 2022)	(Yan et al., 2014; Reuter et al., 2020; Oliveira et al., 2021)

## The application of RPA

### The application of RPA in bacteria detection

The traditional bacterial detection methods were mainly based on the biochemical characteristics of culture method. The culture method took a long time, and some non-culturable bacteria could enter the living state and the bacteria with strict requirements for culture conditions were difficult to detect by the culture method. Therefore, we urgently needed to find a rapid and simple bacterial detection method. Wang et al. established a dual detection biosensor based on RPA and three-segment lateral flow strips to detect Vibrio cholerae and Vibrio vulnificus. This biosensor had the advantages of high sensitivity and specificity, short reaction time, and simple equipment, which was very suitable for detection in primary hospitals and on-site (Wang P et al., 2021). The RPA-lateral flow strips (RPA-LFS) method established by Wang et al. could be used to distinguish capsulated and non-capsulated Haemophilus influenzae, with a detection limit of 1 cfu/μl (Wang et al., 2022). In addition, the RPA-LFS for the detection of Pseudomonas aeruginosa (Yang et al., 2021) and Vibrio parahaemolyticus (Jiang et al., 2020) had also been experimentally verified. Hu et al. developed a detection method combining RPA with polymer flocculation sedimentation. This method could detect as low as 13fg genomic DNA of Staphylococcus aureus and could be directly judged by naked eyes within 20 min (Hu et al., 2020). The double RPA reaction system constructed by Tran et al. could simultaneously detect Staphylococcus aureus and Pseudomonas aeruginosa. It could detect genomic DNA of Staphylococcus aureus as low as 10 fg/reaction and Pseudomonas aeruginosa as 30 fg/reaction (Tran et al., 2022), the sensitivity of Staphylococcus aureus detection was higher than RPA combined with polymer flocculation sedimentation method.

Unlike other bacteria, Vibrio vulnificus could enter a viable but nonculturable state, making it difficult to be detected by conventional methods. Yang et al. established real-time RPA (RT-RPA) for the extracellular metalloproteinase gene of Vibrio vulnificus. This method could be detected only in 2-14 min at 39°C. The detection limit was 17 copies/reaction, and the detection results of clinical samples were 100% consistent with qPCR (Yang et al., 2020). In addition, the vvhA gene could also be used as a specific gene for RT-RPA detection of Vibrio vulnificus (Zhu et al., 2021). Gumaa et al. established RT-RPA and RPA-LFS to detect Brucella. The detection limits of RT-RPA and RPA-LFS were 4 and 6 copies/reaction, respectively (Gumaa et al., 2019). The application of dual RPA could also detect and identify Brucella melitensis and Brucella abortus (Gumaa et al., 2020). Garrido-Maestu et al. established the built-in IAC multiple RT-RPA to detect Listeria monocytogenes. The sensitivity and specificity of the multiple RT-RPA were equivalent to the European reference method (ISO 11290-1), and this method could complete the detection in one working day while using the ISO method took six days (Garrido-Maestu et al., 2020). In addition, Streptococcus pneumoniae (Clancy et al., 2015)and Streptococcus suis serotype 2 (Jiang et al., 2022)could also be quickly detected by RT-RPA.

CRISPR refers to clustered regularly interspaced short palindromic repeats, it was often combined with isothermal amplification methods for nucleic acid detection. An et al. established a one-tube and two-step reaction system to detect Salmonella spp. by combining RPA and Clustered Regularly Interspaced Short Palindromic Repeats associated protein 13a (CRISPR-Cas13a). One-tube and two-step RPA-CRISPR-Cas13a could be detected within 20 min and 45 min, respectively. The detection limits of the two reaction systems were 10^2^ copies and 10^0^ copies, respectively (An et al., 2021). Luo et al. developed an RPA/Cas12a-based system to detect Xanthomonas arboricola pv. prun(Xap). The method could detect as low as 10^−18^ M Xap gDNA with a mini-UV torch, while the sensitivity was 10^-17^ M with LFS (Luo et al., 2021). In addition, the integration of bio-barcode immunoassay, RPA, and CRISPR-Cas12a cleavage into a reaction system could be very sensitive and intuitive to detect Salmonella typhimurium (Cai et al., 2021). In these bacterial detection experiments, RPA showed high specificity, sensitivity, and detection efficiency. Selecting the corresponding RPA results reading method according to different bacteria and detection environments would make bacterial detection more rapid, convenient, and efficient. It could be seen that RPA had its unique advantages in the rapid detection of bacteria.

### The application of RPA in fungi detection

At present, the detection of fungi in medicine was mainly through their morphological and physiological phenotypes. These methods had a long detection time and a low positive detection rate. Cryptococcus neoformans was a conditional pathogen. Most patients had symptoms of central nervous system infection and had high mortality. The common methods to detect Cryptococcus neoformans were ink staining and pathogen culture. The culture method was the gold standard of detection, but the long culture time was not conducive to rapid clinical diagnosis. The ink staining method was limited by the type of specimen, and the positive detection rate was low. Based on this situation, Ma et al. designed high-specific primers and probes for the internal transcribed spacer of Cryptococcus neoformans and established an RPA-LFS for visual and rapid detection of Cryptococcus neoformans/C. gattii. It could detect 0.64 pg of Cryptococcus neoformans genomic DNA, and the sensitivity and specificity were 95.2% and 95.8%, respectively (Ma et al., 2019). When the capsule-associated gene CAP64 of Cryptococcus neoformans/C. gattii was used as the detection target of RPA-LFS, its detection limit was 10cfu/ml or 1 fg/ml(Wang L et al., 2021). Candida albicans was another common clinical pathogenic fungi. Although most people infected with Candida albicans in skin and mucosa were generally not life-threatening, it seriously reduced people’s quality of life and increased people’s economic burden. Wang et al. established RPA-LFS to detect Candida albicans. The detection limit of this method was 1 cfu/reaction, and the detection accuracy was 100%(Wang F et al., 2021a). Leptosphaeria maculans was a highly aggressive fungus that could cause severe phoma stem canker of Brassica napus. After successfully establishing a dual real-time fluorescence RPA to detect L. maculans and L. biglobosa (Lei et al., 2019), Lei et al. further developed an RPA-CRISPR/Cas12a method to detect Leptosphaeria maculans. This method could be completed in 45 min with a minimum detection limit of 4.7 genomic DNA copies (Lei et al., 2022a). With the gradual popularity of RPA, in the field of fungi detection, using visual LFS as the reading method of amplification results was more popular.

### The application of RPA in virus detection

RPA combined with the CRISPR system was widely used in virus detection. Gong et al. developed an integrated trinity test with RPA-CRISPR/Cas12a-fluorescence assay system to detect the respiratory syncytial virus(RSV) A or RSV B. This method could detect the target sequence of 1.38×10^1^ copies/μl and could distinguish RSV A or RSV B infection within 37 min (Gong et al., 2022). Qian et al. established RPA-CRISPR/Cas12a to rapid detection Human norovirus. In this method, CRISPR/Cas12a combined with fluorescence or LFS was used to detect RPA products. The minimum detection limit was 9.65×10^2^ copies/ml, and the detection coincidence rate with qRT-PCR was 98.3%(Qian et al., 2021b). RPA-CRISPR/Cas12a could also detect Human metapneumovirus (Qian et al., 2021a). CRISPR/lwCas13a was another protein that could be used for nucleic acid detection, it had been successfully combined with RPA to detect the African swine fever virus (ASFV) (Ren et al., 2021a) and rabies virus (Ren et al., 2021b). CRISPR/Cas12a and CRISPR/Cas13a systems could be used not only for virus detection alone but also for multiple RPA detection at the same time. Tian et al. developed a dual-gene diagnostic technique for SARS-CoV-2 and ASFV by using the orthogonal collateral cleavage activity of CRISPR/Cas12a and CRISPR/Cas13a system, which showed 100% sensitivity and specificity in the analysis of clinical samples (Tian et al., 2022).

RT-RPA and RPA-LFS methods, which were applied earlier than the RPA-CRISPR, were also widely used in virus detection. Huang et al. successfully developed RT-RPA to detect decapod iridescent virus 1, with a lower detection limit of 2.3×10^1^ copies/reaction (Huang et al., 2022). RPA-LFS could quickly read the test results with the naked eye, so it was very popular in virus detection. Zhang et al. developed RPA-LFS to detect the hepatitis B virus, with a minimum detection limit of 10 copies/reaction and no cross-reaction with other common pathogens (Zhang et al., 2021). RPA-LFS could also jointly detect Epizootic hemorrhagic disease virus and Palyam serogroup viruses, the analytical sensitivity was 7.1 copies/µl and 6.8 copies/µl respectively (Li et al., 2021). RPA-LFS could also be used to detect respiratory syncytial virus (Xu et al., 2020). Because piper yellow mottle virus had two stages of DNA and RNA in its replication cycle, Mohandas et al. established RPA and reverse transcription RPA to detect the virus. The sensitivity of RPA using crude DNA extract as template was equivalent to that of PCR. When using cetyltrimethyl ammonium bromide to extract DNA, the sensitivity of both RPA methods was 10 times higher than that of PCR (Mohandas and Bhat, 2020). Ivanov et al. developed an RPA-LFS method to detect alfalfa mosaic virus and compared two methods for generating labeled RPA amplicons after LFS detection. The results showed that the primer labeled RPA-LFS method could detect 10^3^ copies of RNA within 30 min, and its half-maximal binding concentration was 22 times lower than the probe-dependent RPA-LFS (Ivanov et al., 2021). RPA could also detect tomato apical stunt viroid (Kovalskaya and Hammond, 2022) and barley yellow dwarf virus (Kim et al., 2022) in plants. The application of RPA in virus detection often required reverse transcription first. If reverse transcription and RPA amplification were divided into two steps, there was an increased risk of aerosol generation and contamination.

### The application of RPA in parasite detection

Malaria was a vector infectious disease caused by Plasmodium infection. Early and rapid diagnosis was the key to malaria control. Kersting et al. amplified the 18SrRNA gene fragment of Plasmodium falciparum by RPA-LFS. The detection results were obtained in less than 20 min at 38°C. It could detect the genomic DNA of Plasmodium falciparum as low as 100fg, which was very suitable for on-site detection in remote areas and promoted the progress of global malaria control (Kersting et al., 2014). RPA-LFS could also detect Trichinella spiralis DNA as low as 100 fg, and its sensitivity was about 10 times that of the conventional PCR (Li et al., 2019). When the RPA-LFS method was used to detect Babesia microti, it could detect 0.25 parasite/μl blood, which was 40 times more sensitive than the conventional PCR and had no cross-reactions with DNA of related apicomplexan parasites and their host (Nie et al., 2021). To assess the validity of RPA-LFS for the diagnosis of cutaneous leishmaniasis, Travi et al. used RPA-LFS to test samples from 226 patients. RPA-LFS had a sensitivity of 91.2% and a positive predictive value of 93%. It had potential point of care in cutaneous leishmaniasis endemic areas but may miss positive samples with very low parasite levels (Travi et al., 2021). Molina-Gonzalez et al. successfully used RPA-LFS to detect Giardia duodenalis DNA in stool samples collected in the field. However, the problem of how to extract high-purity DNA from stool samples hindered on-site detection (Molina-Gonzalez et al., 2020). In addition, RPA-LFS had been applied to detect Entamoeba histolytica (Nair et al., 2015) and Trypanosoma evansi (Li Z et al., 2020). RPA-LFS was mainly used for the on-site detection of parasites, and it was important to find simpler methods for high-concentration DNA extraction.

The combination of RPA and real-time fluorescence allowed for real-time detection of the process and had a lower risk of cross-contamination than RPA-LFS. Rostron et al. established RT-RPA targeting the repeat region of Schistosoma haematobium Dra1 genomic. This method could detect 1fg Schistosoma japonicum gDNA, and the results could be obtained within 10 min using a small portable battery-powered tube scanner device (Rostron et al., 2019). RT-RPA could also design primer probes for specific genes of different parasites to establish multiple RT-RPA. Multiple RT-RPA had been successfully applied to the simultaneous rapid detection of Theileria equi and Babesia caballi (Lei et al., 2020). In addition to conventional fluorescent probes, fluorescent dye SYBR Green I combined with RPA was also commonly used in the detection of parasites, such as Plasmodium knowlesi (Lai and Lau, 2020). The SYBR Green I was cheaper than the fluorescent probe. Yu et al. combined RPA with CRISPR/Cas12a to detect Cryptosporidium parvum IId-subtype-family, and the results could be read by the naked eye under blue light or with LFS. This method had robust specificity, showing sensitivities of 1 and 10 copies in pure and complex samples, respectively (Yu et al., 2021). Lei et al. established a portable one-pot assay for Toxoplasma gondii using RPA-CRISPR/Cas2a with a lower limit of detection of 3.3Copes/μl. A portable suitcase was also designed to meet the needs of on-site detection (Lei et al., 2022b). RPA had been successfully applied to detect many parasites, but the application was not yet widespread. Most of the current studies were relatively simple RPA-LFS, real-time fluorescent RPA, etc. Further studies of RPA combined with other methods were relatively rare.

### The application of RPA in drug resistance gene detection

The overuse of antibiotics would lead to bacterial resistance, which would bring great challenges to the diagnosis and treatment of clinical diseases. The monitoring of bacterial drug resistance was of great significance for guiding clinical medication and avoiding or delaying the production of drug-resistant strains. Liu et al. established a 15 µl RPA reaction system to detect carbapenem-resistance genes blaoxa-23 of Acinetobacter baumannii. The test results showed that 90% of the strains showed positive amplification signals, and only 10% of the strains showed negative amplification signals, which was consistent with the results of the PCR (Liu et al., 2020). Methicillin-resistant staphylococci (MRS) was a common drug-resistant strain in the clinic. Srirattakarn et al. established RPA-LFS to monitor MRS, the sensitivity and specificity of RPA-LFS were 92.1% and 100% respectively (Srisrattakarn et al., 2020). RPA-LFS could also be used to detect vancomycin-resistant enterococci (Panpru et al., 2021). Singpanomchai et al. established an allele-specific RPA-SYBR amplification system to detect multidrug-resistant tuberculosis, this method designed specific primers for the alleles of four major mutations rpoB516, rpoB526, rpoB531, and katG315. The experimental results showed that RPA-SYBR had 100% sensitivity and specificity compared with DNA sequencing, and its detection limit for these special mutation sites was 5 ng (Singpanomchai et al., 2021). Wang et al. developed RPA-LFS based on the four most common carbapenemase genes: bla_KPC_, bla_NDM_, bla_OXA-48-like_, and bla_IMP_ for rapid on-site detection of carbapenemase-producing Enterobacterales. The lowest detection limit of this method was 100 fg/reaction (bla_KPC_, bla_NDM_, bla_OXA-48-like_) or 1000 fg/reaction (bla_IMP_), and its sensitivity was 10 times that of PCR (Wang F et al., 2021b). The combination of RPA and real-time fluorescence could also be applied to detect bla_NDM_ gene(Wang X et al., 2021). The rapid, efficient, and simple advantages of RPA could play an important role in the detection of resistance genes. The RPA had the detection ability equivalent to PCR, and sometimes its sensitivity was even higher than that of PCR.

### The application of RPA in detection of genetically modified food

With the development of genetically modified technology and more genetically modified food entering the market, the safety of genetically modified food had attracted more and more attention. At present, there were mainly two kinds of methods for detecting transgenic crops based on protein level and nucleic acid level. The former was limited by protein denaturation, detection reagents, and other reasons and could not meet the detection of large-scale transgenic crops. The PCR method in the latter had high sensitivity and specificity, but PCR required a complex and expensive thermal circulator. Its operation process was complex, which was not suitable for on-site detection. Establishing a rapid convenient detection method would greatly improve the detection efficiency of genetically modified food. Due to the characteristics of isothermal amplification, convenient operation, and fast amplification speed, RPA had been gradually introduced into the field of genetically modified food detection. Li et al. combined single universal primer RPA with LFS to establish an isothermal paper biosensor for multiple detection of genetically modified maize. The biosensor enabled the simultaneous detection of MON810, MON863, and MON89034. The whole analysis process was completed in 30 min without any large instruments, and its detection limit was 50 copies (Li K et al., 2020). Wang et al. combined the advantages of RPA and fluorescence detection, and established a fast, sensitive, specific, and simple MON863 corn field detection platform, with a detection limit of 20 copies (Wang et al., 2020). Different from traditional transgenic technology, RNA interference (RNAi) provided gene silencing at the transcriptional level, which also posed new challenges to traditional detection methods. To improve the stability and amplification efficiency of RPA reaction, Li et al. developed an isothermal fluorescent biosensor based on graphene oxide nanomaterials to enhance RPA, which was used to detect RNAi transgenic plants. The detection limit of this method was 1.5 ng (Li et al., 2022). RPA was convenient and efficient, which was very suitable for on-site transgenic detection in grassroots units, warehouses, and fields. However, only one company sold RPA reagent, which limited its application in large-scale screening of genetically modified food.

### The application of RPA in the detection of SARS-CoV-2

Since the outbreak of SARS-CoV-2, the number of confirmed cases of SARS-CoV-2 continued to rise globally. At present, there was no effective antiviral drug for SARS-CoV-2, so the most important thing for the prevention and control of the SARS-CoV-2 epidemic was still early diagnosis and early isolation. The outbreak of SARS-CoV-2 had made nucleic acid detection well-known. Nowadays, the main methods for detecting SARS-CoV-2 included qRT-PCR, digital PCR, and various isothermal amplification methods. Among them, qRT-PCR was the most widely used method, but PCR had high requirements for equipment, detection condition, and personnel operation. In large-scale detection of pathogens, the speed and simplicity of detection methods were very important, so found a fast and simple method to detect SARS-CoV-2 had become a hot spot. Sun et al. established a double-stranded RPA-LFS detection platform, it could simultaneously realize the rapid visual screening of SARS-CoV-2 and influenza virus, which was conducive to distinguishing patients with SARS-CoV-2 and influenza virus infection with similar clinical symptoms (Sun Y et al., 2022). Shelite et al. established the RPA-LFS for rapid detection of SARS-CoV-2 using the cDNA nucleocapsid gene as the target, with a detection limit of 35.4 viral cDNA nucleocapsid gene copies/μL. The RPA-LFS was 100% consistent with the reverse transcription-qPCR reference test (Shelite et al., 2021). Cherkoui et al. used RPA to simultaneously detect the E gene and RdRp gene of SARS-CoV-2 and designed two optional product detecting methods (real-time fluorescence and test strip) so that the most appropriate method could be selected according to different field environments. The analytical sensitivity of the fluorescence test for the E gene and RdRp gene was 9.5 and 17 RNA copies/reactions respectively, and the analytical sensitivity of the test paper method was 130 RNA copies/reactions (Cherkaoui et al., 2021). A microfluidic-integrated lateral flow RPA for rapid detection of SARS-CoV-2 was established by integrating RPA and LFS detection systems into a single microfluidic chip. The closed microfluidic chip overcame aerosol contamination and the convenient analysis system reduced expensive equipment costs and labor costs (Liu et al., 2021). Choi et al. combined the rkDNA graphene oxide probe system with RPA to detect the SARS-CoV-2 virus within 1h (Choi et al., 2021). The design of RPA as a mobile suitcase laboratory was conducive to the mobile detection of SARS-CoV-2 in the resource-deficient environment (El Wahed et al., 2021). RPA could also monitor SARS-CoV-2 in real-time or at the end point by adding fluorescent dye SYBR Green I. Used SYBR Green I as the fluorescent reporting group did not need to open the tube that may cause aerosol pollution, and this method could observe the test results with the naked eye, and the cost was also lower than RPA-LFS and RT-RPA (Lau et al., 2021).

With the rapid development of the CRISPR system, more and more scholars combined the CRISPR system with RPA to detect SARS-CoV-2. Sun et al. combined RPA with CRISPR/Cas12a to develop a single-tube method to detect SARS-CoV-2. CRISPR/Cas12a detection was carried out in one tube to reduce the liquid transfer step and reduce the risk of aerosol pollution. It could detect SARS-CoV-2 as low as 2.5 copies/μl, and the detection results were 100% consistent with qRT-PCR (Sun et al., 2021). In the single tube reaction, the amplification template loss caused by RPA-CRISPR/Cas12a cleavage would lead to low detection efficiency, Lin et al. found that glycerol additive could significantly improve the detection efficiency of one-pot RPA-CRISPR/Cas12a, and its sensitivity was nearly 100 times higher than that of the method without glycerol. This optimized RPA-CRISPR/Cas12a had been successfully used to detect SARS-CoV-2 and ASFV (Lin et al., 2022). During the establishment of a one-pot detection method based on RPA-CRISPR/cas12a, the development and optimization of methodology could be accelerated by using the statistical design of experiments (Malci et al., 2022). RPA-CRISPR/Cas12a detection system could not only detect SARS-CoV-2 alone but also realize multiple detection of SARS-CoV-2 and other viruses. Sun et al. established the RCD platform based on RPA-CRISPR/cas12a and digital microfluidics. RCD platform showed high sensitivity and specificity and could realize automation and multiplexing. It has been successfully used to detect influenza virus and SARS-CoV-2 (Sun Z et al., 2022).

The 2019 pandemic coronavirus disease generated a huge demand for sensitive and rapid detection of SARS-CoV-2. In recent years, more and more technologies for SARS-CoV-2 detection combined with RPA had been developed. Based on the high peroxidase-like activity of the FeS2 nanozymes, Meng et al. combined RPA and FeS2 nanoenzyme strips to achieve nucleic acid amplification and subsequent colorimetric signal enhancement. The method had a detection limit of 200 copies/ml for SARS-CoV-2 (Meng et al., 2022). Hu et al. established a light-controlled CRISPR-RPA method to detect SARS-CoV-2. The crRNA was designed to be temporarily inactivated and the CRISPR-Cas12a detection system was activated under rapid light irradiation after the RPA reaction was completed. RPA and CRISPR/Cas12 system integrated into a completely closed tube to avoid the risk of contamination. Compared with the conventional OnePot detection, the sensitivity was improved by more than two orders of magnitude (Hu et al., 2022). Park et al. developed the first digitization-enhanced CRISPR/Cas-assisted one-pot virus detection (deCOViD) method and applied it to detect SARS-CoV-2. The deCOViD was based on RPA-CRISPR/Cas12a, which enabled qualitative detection in 15 min and quantitative detection in 30 min. It was highly sensitive and could detect down to 1 genome equivalent (GE) µL^−1^ of SARS-CoV-2 RNA and 20 GE µL^−1^ of heat-inactivated SARS-CoV-2. This method was one of the fastest and most sensitive SARS-CoV-2 detection methods based on CRISPR/Cas (Park et al., 2021). Some new methods combined with RPA were not very mature at present, but they showed great advantages in improving detection sensitivity. In conclusion, RPA had low requirements for equipment and field detection environment and was very suitable for on-site screening of SARS-CoV-2.

## Conclusion

As a technology that could complete nucleic acid detection at 37-42°C, RPA had been greatly developed in recent years. Compared with PCR and other isothermal amplification methods, RPA had the advantages of simple operation, fast reaction speed, and low requirements for equipment. In terms of sensitivity, RPA could detect trace-level nucleic acid in samples at the lowest level. In some studies, RPA could even detect low concentrations of DNA that could not be detected by PCR. In terms of specificity, RPA could identify and amplify target genes from the genomic DNA of different species and specimen types. It had a strong anti-interference ability, the detection results were highly consistent with PCR. In addition, RPA could run at 37-42°C without complex temperature control equipment, which was very suitable for on-site detection in low resource environment. And RPA could get test results within 20 min, which was conducive to large-scale screening and rapid detection of samples. The mild reaction conditions and high amplification efficiency of RPA made it very suitable for rapid clinical diagnosis, food detection, epidemic prevention and control, industrial application, and on-site real-time detection. Especially after the outbreak of SARS-CoV-2, nucleic acid testing had become normalized, and the scale of testing had become larger and larger. A rapid, accurate, and efficient detection method would play an important role in epidemic prevention and control. To make RPA more efficient and convenient, people had taken various optimization and improvement measures in the amplification system and detection result reading system of RPA, such as adding glycerol that could improve the detection sensitivity, simple visual product detecting method, portable suitcase laboratory, and so on. Of course, as an emerging method with a short development time, RPA also had certain limitations. For example, no specialized software had been developed for the design of RPA primers, and only PCR software could be used for design and screening. The primers of RPA were longer than PCR primers. Sometimes the RPA could amplify the corresponding fragments with PCR primers, but it might not achieve the optimal amplification effect. At present, RPA technology has not been widely used mainly because it is not an open technology and is only used for scientific research. In addition, only one company sells RPA kit, which is expensive and costable for the detection of large-scale samples. Although RPA technology still has some limitations, it is expected to become the mainstream technology of nucleic acid amplification in the future by further exploring it and amplifying its advantages.

## Author contributions

MT: Collect data, write and revise articles; CL: Collect information and assist in writing articles; XY: Guidance; ZZ: Article revision; LL: Guidance; GW: Review and modification of final draft. All authors contributed to the article and approved the submitted version.

## Funding

Our research was granted by Natural Science Foundation of China (82260418), Natural Science Foundation of Guangxi (2019JJB140034), First Batch of High-level Talent Scientific Research Projects of the Affiliated Hospital of Youjiang Medical University for Nationalities (R202011701) and Baise Scientific Research and Technology Development Project (20193121).

## Conflict of interest

The authors declare that the research was conducted in the absence of any commercial or financial relationships that could be construed as a potential conflict of interest.

## Publisher’s note

All claims expressed in this article are solely those of the authors and do not necessarily represent those of their affiliated organizations, or those of the publisher, the editors and the reviewers. Any product that may be evaluated in this article, or claim that may be made by its manufacturer, is not guaranteed or endorsed by the publisher.
